# Being stuck in a vice: The process of coping with severe depression in late life

**DOI:** 10.3402/qhw.v10.27187

**Published:** 2015-06-26

**Authors:** Guro Hanevold Bjørkløf, Marit Kirkevold, Knut Engedal, Geir Selbæk, Anne-Sofie Helvik

**Affiliations:** 1 Norwegian National Advisory Unit on Ageing and Health, Vestfold Hospital Trust, Tønsberg, Norway; 2 Department for Mental Health Research and Development, Division for Mental health and addiction, Vestre Viken Hospital Trust, Lier, Norway; 3 Faculty of Medicine, University of Oslo, Oslo, Norway; 4 Research Centre of Old Age Psychiatry, Innlandet Hospital Trust, Ottestad, Norway; 5 Department of Public Health and General Practice, Faculty of Medicine, Norwegian University of Science and Technology (NTNU), Trondheim, Norway; 6 St Olav's University Hospital, Trondheim, Norway

**Keywords:** Lived experience, depression, older people, coping

## Abstract

Articles describing older persons’ experiences of coping with severe depression are, to our knowledge, lacking. This article is methodologically grounded in phenomenological hermeneutics, inspired by Paul Ricoeur, and applies a descriptive design with in-depth interviews for producing the data. We included 18 older persons, 13 women and 5 men, with a mean age of 77.9 years, depressed to a severe or moderate degree, 1–2 weeks after admission to a hospital for treatment of depression. We found the metaphor “being in a vice” to capture the essence of meaning from the participants’ stories, and can be understood as being stuck in an immensely painful existence entirely dominated by depression in late life. This is the first article where coping in older men and women experiencing the most severe phase of depression is explored.

Older people constitute the fastest growing part of the population and 1–5% are found to suffer from a severe depressive disorder (Cole & Yaffe, 1996; Hasin, Goodwin, Stinson, & Grant, 2005). Because severe depression in late life is associated with increased risk of morbidity and mortality and decreased functioning and quality of life (Blazer, 2003), the future cost to the patient, the caregiver, and the health services will be significant (Mathers, Fat & Boerma, 2008). The development of depression in late life has several causes (Brodaty et al., 2001; Heun, Papassotiropoulos, Jessen, Maier, & Breitner, 2001). It is shown that late-onset depression frequently occurs in the context of somatic illnesses (Engum, 2007; Krishnan, 2002; Park et al., 2007) and also represents a prodromal phase of dementia (Alexopoulos, 2005). Others highlight that depressive disorders in late life can be understood as individual responses to unique configurations of stressful life events during a lifetime (Fiske, Wetherell, & Gatz, 2009). In a meta-analysis, bereavement, sleep disturbance, disability, prior depression, and being female were significant risk factors for the development of depression in late life (Cole & Yaffe, 1996). Remission rates of depression show little difference between middle-aged and older persons, but relapse rates appear higher in older people. A review on prognostic factors of depression in older people in a community setting found persistence of depression to be associated with baseline depression levels, older age, somatic comorbidity, functional limitations, and external locus of control (Licht-Strunk, Beekman, De Haan, & Van Marwijk, 2009). Furthermore, a review of the literature regarding studies of older peoples’ depression and different concepts of coping also revealed that more frequent use of active and problem-focused coping, higher internal control, a higher sense of coherence, and more religious coping were strongly related to lower levels of depressive symptoms (Authors). Coping is thus well documented to be a relevant factor to the understanding of depression in late life.

Some studies have reported that the way older men and women understand and explain their depression is found to affect how they cope with their condition in primary care settings (Kadam, Croft, McLeod, & Hutchinson, 2001; Switzer, Wittink, Karsch, & Barg, 2006). Also, it is found that older adults of both sexes and from different ethnic groups use different strategies of coping regarding depressive symptoms, that is, spiritual strategies were important in addition to support from the family (Barusch & Wilby, 2010; Bennett, Smith, & Hughes, 2005; Jensen & Munk, 2010; Lawrence et al., 2006). Among nursing home residents, an article revealed how aspects of coping, such as loss of control and autonomy, were relevant for the residents’ experiences of negative emotions (Choi, Marti, Bruce, & Hegel, 2013).

For some time, severely depressed and psychiatric hospitalized older persons have been considered as too vulnerable to be interviewed (Usher & Holmes, 1997). Wendy Moyle (2002) by doing unstructured interviews of seven severely depressed hospitalized men and women about their experiences of being cared for during hospitalization, however, revealed how they gave valuable descriptions of their experiences.

Furthermore, Pollitt and O'Connor interviewed 50 older persons about their experiences of being admitted to an old-age psychiatric ward when suffering from severe depression (Pollitt & O’ Connor, 2007). The interviews were conducted 3–26 weeks after discharge from the hospital in the participants’ homes and revealed that being hospitalized was experienced as traumatic to some, but in light of the recovery, the experiences were re-evaluated. An article by Allan and Dixon also describes four previously depressed women above 65 years of age, recruited from an outpatient clinic, feeling overwhelmed by their emotional condition, expressed as fear of losing control by the struggle of everyday life (Allan & Dixon, 2009). These women were, however, not depressed during the interviews that took place in their own homes. Hedelin and Strandmark, in their study, included five women aged between 75 and 92 years with depression (Hedelin & Strandmark, 2001). These women told of experiences of specific life events or of a series of traumatic events related to close family members that were considered to play a major role in how they understood their depressive symptoms (Hedelin & Strandmark, 2001). Nevertheless, the women in these two phenomenological studies (Allan & Dixon, 2009; Hedelin & Strandmark, 2001) were neither hospitalized nor did they suffer from severe depression during the interviews; the studies were retrospective and coping *per se* was not explored. Thus, we have not found any article where older men and women with moderate or severe depression have described their understanding and experiences of coping in the midst of their crisis.

## Aim

The aim was to reach a deeper understanding of how older women and men experience severe depression and cope with their situation.

## Method

To reveal the essence of meaning from the transcribed narratives of the participating men and women, we chose a methodology grounded in phenomenological hermeneutics inspired by Geanellos (2000), Lindseth and Norberg (2004), and Ricoeur (1974a, 1981a). Phenomenological hermeneutics seeks to describe and interpret the experiences and meanings of persons’ lived experiences of particular phenomena and situations.

### The participants

Eighteen patients (13 females and 5 men) from both rural areas and cities, hospitalized in a psychogeriatric hospital unit in Norway in 2010 and 2011, were included. They were recruited from a multicentre longitudinal study of older people with depression. The patients ranged in age from 64 to 89 years, and the mean age was 77.9 (SD 7.9) years. Of the18 patients, 3 were diagnosed with moderate and 15 were diagnosed with severe depressive disorder according to WHO's international classification of diseases and related health problems, 10th edition (ICD-10) (WHO, 1993). Eleven patients experienced their first depressive episode at the time of recruitment, and seven patients suffered from a recurrent depressive disorder, including one person diagnosed with a bipolar affective disorder. Four of the patients with recurrent depression had an early-onset depressive disorder, that is, the first episode occurred prior to the age of 65. Two of these patients had suffered from two earlier episodes, and the other two patients with early-onset depression had more than five earlier episodes. Of the three patients with recurrent depression and late-onset depressive disorder, two had suffered from two earlier episodes and one from five earlier episodes. All patients, except for four persons, were evaluated to have either a minor cognitive impairment, or were cognitively intact during the interview. Four persons had a diagnosis of dementia. The score on the Mini Mental State Examination—Norwegian Revised Version was ≥22 in all participants (Folstein, Folstein, & McHugh, 1975). All but three patients (who resided in a service home) resided in their own apartments and six lived with their spouses. Two patients were divorced, nine were widows or widowers, and one woman was single. Five of the patients had some comorbidity that affected their level of functioning and quality of life, including one person with Parkinson's disease, one with chronic pain, two with eye diseases, and one person with an anxiety disorder.

### Procedure

Patients aged 60 years and above, diagnosed with a depressive episode or disorder according to ICD-10 criteria for research, were eligible for inclusion. Evaluation of inclusion and exclusion criteria and the diagnostic assessment were done by psychiatrists and psychologists experienced in geriatric psychiatry and geriatric psychology. The exclusion criteria were to have a severe cognitive impairment, evaluated by a score of ≤22 on the Mini Mental State Examination—Norwegian Revised Version (MMSE-NR) (Engedal, Haugen, Gilje, & Laake, 1988; Folstein et al., 1975; Strobel & Engedal, 2008), or having severe aphasia or a life-threatening condition. When the inclusion criteria with regard to MMSE scores were met, the ability of the patient to understand the purpose of the study and to give oral and written consent to participate in the study was assessed. All patients were interviewed between 7 and 14 days after being admitted to a psychogeriatric hospital unit with 10 beds.

Prior to invitation to participation in the study, the patients’ competence to consent was evaluated by the medical staff in charge. Thereafter, the patients were invited to participate by the clinical staff. Information about the qualitative study was given in oral and written form both to the patients and to their next of kin before they were asked to give a written consent to participation. The research project was approved by the Regional Committee for Ethics in Medical Research in South-Eastern Norway and the Data Inspectorate (REK 2009/1774), and was carried out according to the World Medical Association Declaration of Helsinki (WMA, 2001).

### The interviews

The interviews were conducted in a psychogeriatric hospital setting and took place either in the participant's room if they preferred, or in an office connected to the unit. The setting of the interview was adapted to participant wishes as much as possible. If a participant was perceived to be in discomfort during the interview, either by the researcher or brought up by the participant, the researcher could suggest ending or pausing the interview, or in other ways make changes according to the participant's wishes (Kvale, 1996). The participant and researcher had dialogues in response to the researcher asking open-ended questions. Thus the participants were invited to tell their stories. The interviewer used a thematic interview guide (Kvale, 1996; Moyle, 2002). The following main questions were asked: “How do you experience being depressed?”, “How do you understand your condition and situation in life right now?”, and “How do you cope with the situation?” The researcher aimed to follow the participants’ stories but also probed to encourage more in-depth descriptions and validate his or her interpretations to understand their experiences and thoughts. The interview was ended by the participant, on the suggestion of the researcher continuously evaluating the condition of the participant, or when no new information was revealed. The interviews lasted between 30 min and 2 h, and were recorded by an iPod. When the researchers found that the descriptions of the participants’ experiences from all the interviews became repetitive and no new nuances were revealed, the inclusion of new participants ended (Kvale, 1996). If a participant wanted to read the transcript afterwards, he or she was given the transcript and could make comments. This was done to assure the participants that their stories were recognizable to them after transformation from spoken to written words. The researcher, however, considered these comments as additional information and not as part of the interviews and informed the participants of this approach (Ricoeur, 1974a).

### Analyses of the material

To extract the essence of meaning from their experiences of coping with severe depression, the audiotaped interviews were transcribed verbatim from the iPod into written text as accurately as possible, including pauses, and non-verbal sounds. During this transformation, the written text was considered “objectified” from the participant by the researcher processing the information and unavoidably interpreting the spoken words and sounds herself or himself before writing it down into readable text (Geanellos, 2000). Interpretation can thus be understood as a result of interplay between the interpretation and the interpreter (Geanellos, 2000; Ricoeur, 1974a, 1981a). The researcher as an interpreter is contributing to the extracted meaning and final results extracted from the participants’ stories (Geanellos, 2000). It is important to highlight the researchers’ pre-understandings. The initial analyses and categorizations of the material included three researchers (authors), one clinical psychologist and two nurses with different clinical backgrounds and research training. The last two researchers, two medical doctors with old age psychiatry backgrounds, participated in the latter part of the analysis and interpretation of the material. The researchers’ contributions during the interview and interpretative process were continuously reflected upon in a reflexive journal, together with the participants’ reactions towards the researcher and the situation regarding how the interview was arranged.

The following steps of analyses were conducted: (1) *Naïve reading*. The audiotapes were listened to and the transcripts were read through several times to gain an overall understanding of what the participants narrated, the atmosphere of the interview, and the essence of the meanings in the stories of the participants. Condensation of the participants’ stories was also initially performed to bring their stories further into “relief” and to bring forward the essence of the stories. This was important because the participants had problems with forming sentences and needed time to express themselves verbally. These impressions from every transcript were written down and summarized as an initial interpretation of the text (not shown). (2) *Structural analysis* was conducted by first dividing the text into meaning units, labelling words, phrases, and sentences according to their meaning. Units of text revealing similarities and variations of experiences and meaning from across the transcripts were then grouped into larger units of text, forming categories and emerging themes. The categories and emergent themes were compared to each other, revealing further variety and density of meaning. Themes were then read as wholes and different sub-themes emerged related to every main theme. (3) Through the process of *comprehensive understanding*, the researchers summarized and reflected upon the main themes and sub-themes to understand the analysed and complex text as a whole and to see the essence of meaning revealed. By returning to the original transcripts and the initial naïve understanding, again as naïve and open-mindedly as possible, the themes and sub-themes were read in relation to their original context to see if the abstracted and condensed units of text still reflected the meanings from the original transcripts.

### Trustworthiness

The first author worked as a psychologist at the unit where she conducted all the interviews. It was crucial not to have any formal role in relation to the participating patients and by this to put a participant under pressure during inclusion or during the interviews. This conflicting double role was reflected upon from the beginning and was continuously discussed in a reflexive journal and with the other researchers and colleagues at the hospital unit during the entire period of the project (Tong, Sainsbury, & Craig, 2007). In addition to written information, oral information was given about the study, the roles of the researcher, and confidentiality, both before, during, and after the interviews. Techniques such as meta-communication were applied to assure and make roles and the context of the questions more clear between the participants and the researcher: “I'm asking this as a researcher.” The researcher also asked for renewed consent if the participants during the interview entered sensitive issues: “Is it O.K. for you to talk about this?” This renewed consent was again requested after the interview was finished. The researcher also told that what the participating patient was choosing to say was not written in the journal, or told to other clinical staff, but was considered as research data. If critical information that the researcher found important considering the participants health was told by the participant, the researcher would ask for permission to forward this to the clinicians. The participants were also invited to comment on their transcript. Furthermore, also being an experienced clinical psychologist influenced the pre-understandings of the researcher and made it challenging to keep enough distance to the participant to be able to see more than the “depressed patient.” It was crucial to be able to bring to consciousness these issues and analyse the material with an independent and experienced group of researchers. By self-reflection and disclosure, contributions from the researcher to the participants and the interview process, were highlighted. Methodologically, being experienced as a clinical psychologist might also have contributed to the researcher's feeling of security when talking with older persons suffering from a severe depressive disorder. This might also have made it possible to conduct interviews with severely depressed older persons during hospitalization.

## Results

The depressed patients were positive about being invited to talk about their experiences. Although the themes are based on all the interviews, some of the participants were able to describe their experiences and situation in greater detail than others and these interviews are more often referred to, regardless of the severity of their depressive condition. Three main themes were identified from the material capturing the participants’ experiences of depression and how to cope with it: (1) “Terrible suffering,” (2) “Being stuck,” and (3) “Why did this happen?” The first two themes, described in all the interviews, were continuously and constantly present during the interviews. We found these two themes to capture the patients’ intense and agonizing mental state and efforts to cope. The third theme revealed the participants’ own understanding or explanation of the reasons and context of their experienced agonizing existence. All the main themes were strongly related to each other. The researchers found the metaphor “being in a vice” a useful picture to capture the essential meaning across the main themes of the participants’ expressed experiences of coping with moderate and severe depression. In the following, we describe each of the main themes in detail, referring to the main subthemes and using representative quotes to illustrate the main findings.

### Terrible suffering

Suffering from depression was described as a terribly agonizing and painful condition. Being in this state was described as a highly intense and almost unbearable experience. The descriptions were mainly of (1) “an overwhelming feeling of restlessness,” of (2) “aches and pains all over the body,” and of (3) “being drained of energy.”

#### An overwhelming feeling of restlessness

Descriptions of anxiety and unrest were predominant among the participants. They described themselves as terribly scared, running on a high gear overwhelmed by a feeling of restlessness, afraid of losing control, becoming panic ridden and not being able to restrain themselves. Bertha was 74 years old. She cared for her husband until he died 6 months ago. She took care of all the paperwork and practical issues in relation to his funeral, and she looked after the house and garden. At some point she felt totally worn out. She could not find time or a place to rest. Finally she was admitted to the acute psychiatric ward at the hospital with a severe depressive episode. She described her overwhelming restlessness in the following way:

Just wandered, and wandered and wandered. Went about like a dog in a cage … couldn't sit and eat. I stood and … stood and ate actually … don't remember what controlled my legs … went like drumsticks. Don't know what is steering. That is what is scaring, that you don't manage to hold your legs still. You will … and … and don't manage to get control of your nerves. It's … It's like a motor inside that has started and that you have no control of.

The intense feeling of restlessness acted for some of the participants like a two-stage rocket where the experience itself led to even more fear. This experience was described as a fear of becoming seriously ill or of dying. For some of the participants, this eventually led to panic and deep despair. Holly was an 89-year-old widow suffering from severe depression. She became depressed after her only son and daughter-in-law did not invite her for Christmas holiday. She described how an overwhelming restlessness ended in desperation:

It's an unrest … a great restlessness inside me … don't know what to do. I became desperate. I knew something had to happen. The doctor I had, she had tried ten different tablets … and it did not help … it just became worse and worse.

#### Aches and pains all over the body

The participants described a number of bodily discomforts and painful conditions connected to being depressed. These included physical pain and tension in muscles around the jaws, neck, head, shoulders, chest, or stomach. During the interviews, the participants moaned and sighed. Some also asked for the interview to be paused or stopped because it was painful to sit for some time, in addition to the emotional pain and bodily discomfort they described when speaking about themselves and their lives. Some participants also experienced their physical condition as their main problem, and had several referrals to specialists for different physical examinations thinking they were suffering from a physical condition. Henny was a single woman 70 years of age. She used to work in business and was now retired. She had some friends in a club, but her family lived far from her:

No, it's terrible with all this pain … and isolation. I ended up sitting in my sofa with a feeling of being stressed. Then my neck started to ache, head started to ache, shoulders and back. It aches all those places … and it's terribly painful in my jaws and eyes. It is stress and maybe that I have been doing too much crosswords. Then I went to my doctor again and he said: ‘Well, now you have been examined all over, and there is nothing more we can do for you’. Then he sent me to a psychologist and I said that was not what I needed. I need something else. I need help to get started and to get rid of all the pain.

#### Drained of energy

Many felt exhausted and drained of energy. Daily routines became a heavy burden to fulfil even though such activities previously had not caused any burden. Some were bedridden and not able to maintain hygiene or nutrition. Experiencing this condition was also described as being close to dying. Hans was 74 years old. He was a widower and suffered from a bipolar disorder. He was admitted to hospital because of the development of a severe depressive episode:

I just slept away and … I was sad and … that is the terrible thing with being depressed. You become powerless and … you try to stay active … to eat … I try … it's not much to do with these things … I will die.

### Being stuck

All participants gave descriptions of how they had tried previously successful strategies to reduce discomfort and unrest. Many tried to develop new strategies of coping with the emerging depressive symptoms. They used to manage life alone or together with their family, but eventually could no longer sustain a normal standard of living, then sought help from their general practitioner (GP) and were eventually admitted to a psychogeriatric hospital. A few wanted to stay at home and were admitted against their will because of a life-threatening health status. Becoming depressed led the participants to feel they were stuck. “Being stuck” was associated with experienced helplessness, powerlessness, and perplexity, summarized by the subthemes: (1) “I lost my way,” (2) “Can't pull myself together,” and (3) “Giving in.”

#### I lost my way

Most participants had prior experiences and reflections about strategies helping to reduce depressive symptoms, but now experienced that such relief that formerly ensured was gone and they had lost their way out of their misery. Some had learned techniques to reduce the unrest, others had previously coped by using physical, social, or religious activities. Others again felt in control when being able to fulfil their daily routines. At some point these strategies were no longer efficient, and this was a devastating experience. They tried even harder or sought more help from their families or friends, stopped using their GP and approached other GPs, or approached alternative medicine. These participants described how they did not know what to do anymore and ended up feeling stuck, helpless, and with a rising feeling of anxiety and panic. For example from Holly:

Don't know what to do …. I used to go out and visit friends … ask them what they do … and if I could pay them a visit … yes, that helped … but now the feeling has gone … before it could feel so good when the phone rang, but now … I'm longing so much … when I hear a voice.

#### Can't pull myself together

Descriptions of powerlessness and frustration were given by participants who used to cope by being active doers, who had worked their entire life, and used to feel powerful enough to make a change if that was needed. By fixing the roof, going for a walk, or cleaning the house, they felt useful and good about themselves. They now felt unable to act. Strategies were out of reach because of the struggle with initiative and anxiety, and this felt even more frustrating. This further affected how they perceived themselves and many did not manage to act according to their anticipations about themselves anymore. Feelings of guilt and shame were described when broaching this issue, and of not being able to “pull myself together.” This contributed, for some of the participants, to a feeling of alienation. Some could see how they had physically changed in their face and some experienced that their personality was altered, that they were not themselves anymore, and acted in unusual ways. In addition, some described that their lack of initiative or ability to implement strategies “got in the way” of being able to recover or receive treatment for their depressive disorder. Not being able to contribute actively in the process of healing and treatment contributed to a feeling of being a failure, of being weak, and being a burden; “to lose” in the words of one. Peter, 74 years of age, collapsed after enduring efforts of being strong and active, trying to cope with being in an utmost painful existence when caring for his dying wife. He used his familiar strategy of coping, being an active doer, but his active efforts of coping did not bring any change to his painful situation. His wife eventually died and he developed a severe depressive episode:

I thought I could cope with this … through … until she died. I managed to get energy to pull myself together … to function together with her … the laundry and all that. To take her out for a walk … I could … at the end I was down at the bottom.

#### Giving in

Some eventually did not try to alter or control their condition and just resigned. They felt there was no meaning left in life and, without knowing what to do, they kept themselves isolated from those who might have given them support. For some of the participants, entering this state of their depressive condition ended in life-threatening somatic conditions after not being able to care for themselves, after suicide attempts, or after simply not communicating the need for help. Others reflected on their state as a phase of dying, or felt that they would rather die than suffer as they did. Annie was 89 years old and had developed a severe depressive episode after a fall. She thought she would lose her ability to do gymnastics with her training group, an activity most meaningful to her. She resigned, convinced that she was too old to recover:

Annie (A): When you … are at my age, it is not that easy to get up on your feet again … and I thought that when I went out there for the second time [to hospital], I would never manage to do so. There were so many thoughts inside your head …

Researcher (R): What thoughts could that be?

A: Well … that I rather could end it all than have to experience this.

R: You would rather die?

A: I would rather die than to live like this.

### Why did this happen?

Among the participants, some expressed they did not comprehend at all why they had ended up in such a painful existence, whereas others sought to explain their current condition and situation in relation to the life they had lived, and described that they had experienced heavy demands or events that eventually resulted in acute crises. These experiences were either one major event or described as accumulative events or burdens over time. Finally, life became overwhelming. Their experiences are described as (1) “too much of a burden from caring,” (2) “being left alone,” or as (3) “fear of becoming too much of a burden to others.”

#### Too much of a burden from caring

About half of the participants described themselves as being in a caregiver relationship that had developed into a burden too heavy to bear, either in caring for dying spouses or spouses with a physical disability or dementia. They also cared for children being abused by members of their families; some cared for children addicted to drugs, alcohol, or who had a physical illness. Some experiences were from a long time ago and some were traumatic, that is, being stabbed with a knife, finding a husband shot dead. To be a caregiver was a natural role to take, but now they felt overwhelmed by never being able to work through it or to be able to relax. They described eventually developing a depressive episode from all the grief, worry, and exhaustion. They seldom asked for help for themselves, but were used to standing up for another person's needs. Eva developed a severe depressive episode for the first time in her life at the age of 78. She was a widow, who had cared for the needing people in her family all her life, but when her son divorced, she was overwhelmed and her depression surfaced:

Then my son said to me, ‘Is this because of me?’ No, I said. It's just that it has been so many things throughout so many years … that I have just shuffled it and shuffled it away. It just had to go! … and ever since … it has been so many things in the family … it has been like that all my life, I think.

#### Being left alone

Others felt they lived isolated lives due to loneliness, physical illness, and being dependent on care themselves. They had in common an experience of being left alone. The help they received was felt as inadequate, and they described being bitter, suspicious, or hostile. They all felt troublesome to their social surroundings, misunderstood, and that their quality of life was low. They were in emotional pain and suffered because they experienced too little external support and attention from their spouses, children, a girlfriend, and from the health care system. Harry (H), an 84-year-old man living with his wife, had developed a severe depressive episode. He was feeling jealous, suspicious, and angry with his wife:

H: When you don't manage to find things to be right as it is now, then you probably get more easily irritated. In addition, I am getting older and more jealous.

Researcher: How do you feel about that?

H: [Laughs] Well, it's not a good feeling! … it has more or less added up into … She is very charming towards other people. Has a lot to say and can make a joke and things like that … so then, but not directly … a charming woman … it's natural that another part, the way I see it, finds this attractive and finds her attractive and so on … I can't find any direct example, but it is probably a part of the background.

#### Fear of becoming too much of a burden to others

Some did not give an explanation or understanding of their misery, but rather blamed themselves for how they felt. The participants in this group were all divorced or widows living alone and had become increasingly dependent on care because of physical illness. They had in common a deep fear of becoming dependent on care and they all wanted to live without receiving external help. They did not want to become a burden to anyone, most importantly, to the members of their families. They used to care for spouses or children themselves earlier in life. They did not associate any negative events in life to their current depressive condition. On the contrary, they were blaming themselves for their misery and for not managing to control themselves and their unhappiness more. Two of the women in this group had tried to commit suicide. Rita was 88 years old and lived alone as a widow with no children, but she had a niece that was very dear to her. She was overwhelmed by guilt and blamed herself for ending up in such a severe condition and for having caused disappointment to her niece:

I blame myself for having … that is, I'm blaming myself … that I have put myself into this situation I'm in now … yes … self-blaming you could say … [whisper] … no … I'm totally … no, I … that I was going to manage myself without … that I wasn't going to manage the expenses of my housing and … yes … that I was bothering … my family. They say they don't … that there is no reason … for … no, that I have disappointed them in some way …

#### Being in a vice

We found “being in a vice” as a useful metaphor to capture the overall understanding of the participants’ lived experiences of coping with moderate to severe depression. A vice is a mechanical screw apparatus that can hold “something” in a fixed position by two jaws that can be either tightened or loosened by the screw. The stories revealed experiences of being locked or fixed in a most painful existence by forces that participants could do very little to loosen the grip of. No matter how they approached their situations, how hard they tried, or what strategies they sought, they were stuck and not able to alter their conditions.

## Discussion

Our main finding was that older men and women being admitted to hospital with depression suffer greatly because of the experience of being stuck in an immensely painful existence. They strive to stay alive and experience a “terrible suffering.” The old men and women felt tormented by restlessness and unease, felt aches and pains all over, and were drained of energy. Such feelings can be linked to a severe multidimensional and unbearable suffering affecting physical, mental, social, and spiritual aspects (Hedelin & Strandmark, 2001). Furthermore, the participants in the present study found no relief from the burden of depression, ending up overwhelmed by an experience of “being stuck.” The old men and women described despair, powerlessness, and perplexity; they could not pull themselves together and finally gave in. These findings are comparable to findings described in another paper of older, depressed women that struggled to initiate the simplest of tasks and were unable to force themselves to do it (Allan & Dixon, 2009). Furthermore, some similarities with our results can be found in a study exploring depression in younger African American men that experienced a mental “breakdown” followed by a severe depressive condition (Bryant-Bedell & Waite, 2010). Our findings also show similarities with findings in studies of older persons experiencing loneliness. These participants felt trapped and also unable to overcome and cope with their situation themselves (Hauge & Kirkevold, 2010, 2012).

Many of the participants in the present study were perplexed and wondered, “Why did this happen?” They told of former recourses and strategies of coping and that these resources and strategies were no longer available, were not efficient, or no longer sufficient. They also told stories of bearing too great a burden from caring for other persons in life, of being left alone, or a fear of becoming a burden to others. Other studies, including younger adults have shown how different ways to understand major depression were used as a background to cope with their depressive illness differently (Nunstedt, Nilsson, Skärsater, & Kylen, 2012). In the present study, however, we did not find that the participants were able to make necessary changes and to act even though some had made up their minds about what might have caused or triggered their severe depressive conditions. In addition, in our study, the participants’ relationships were sometimes complicated by the caregiving participants being abused by the person receiving care, or by experiencing traumatic events within their family. This finding corresponds with those of Hedelin interviewing older women describing a specific life situation or series of traumatic events central to their depression (Hedelin & Strandmark, 2001). Thus, there is reason to believe that this group of older men and women suffering from depression is an especially vulnerable group. In contrast to the experiences reported by younger adults and persons with less severe depression (Amini, Negarandeh, Cheraghi, & Eftekhar, 2013; Brintnell, Sommer, Kuncoro, Setiawan, & Bailey, 2013; Peden, 2000; Poslusny, 2000; Skärseter, Dencker, Bergbom, Häggstrom, & Fridlund, 2003; Skärseter, Dencker, Häggstrom, & Fridlund, 2003; Switzer et al., 2006), the older men and women of the present study being interviewed in the midst of their crisis seemed unable to find ways to effectively deal with their situation and relieve their emotional pain. They were in this respect at a different stage in the process of coping, a phase where few prospects of healing or hope were found without considerable support from their social surroundings. They also had a physical, social, and cognitive vulnerability due to higher age, comorbidity, and the severity of their depression, making their process of coping even less resourceful. Nevertheless, the older participants were able to tell their stories. By inviting the older men and women to present their own perspective of their current condition and situation in life, this study has shown that essences of meanings can be revealed even in the midst of a depressive crisis, by using a phenomenological approach. Through a process of storytelling, older depressed men and women may also uncover alternative, more positive narratives or empowering stories of how they have mastered situations in the past (Frank, 1998; Kraus, 2007; Taylor, 2007). This may enhance reflection of the patient's abilities and competence, and be a way to find new solutions and hope. Narrative therapy offered by professional health caregivers in a supportive context may therefore be a psychological intervention, especially suitable to this group of patients.

### Methodological considerations

To our knowledge, the present phenomenological article is the first to explore the experience of coping with severe depression in hospitalized older men and women, shortly after admission to a psychogeriatric hospital unit. The process of coping is thus explored in the present and not retrospectively, which we consider as the strength of this study. The authors of this study consisted of researchers representing different health-related professions and pre-understandings, thus contributing to the analyses and discussions of the material becoming more thorough and broad. Due to the severity of some of the participants’ conditions, and to problems with cognitive functioning and reduced speed mainly caused by the severity of their depressive symptoms, some participants had difficulties forming sentences, making them less able to tell a coherent story. Repetitive interviews with the participants at the time of admission may have enriched the material of this article. The researcher, being a younger female, may also have limited what the older men and women chose to tell and in what ways they would express themselves.

Our findings were revealed through interpretation of the texts. Thus, our own contributions due to our pre-understandings make us ourselves part of the process of interpretation (Ricoeur, 1974a, 1981a). The results reflect the essence of the meanings initially expressed by the participants during the interviews, but in a processed manner more abstracted and generalized. Even though contributing with different pre-understandings, by reading and interpreting the text individually and in discussions, the researchers reached consensus on themes and subthemes from the interviews. We find we have been able to ensure different aspects and interpretations of the text were included, and have reached a thorough enough understanding of the participants’ stories to obtain a more profound understanding of the phenomena under study.

## Conclusion

We found great suffering, overwhelming experiences from burdens in life, and a perplexity as to why this had struck the older men and women in this study, as our main findings. The metaphor “being in a vice” was chosen by the researchers to reflect the participant's experiences from the totality of all their stories.

Being stuck in helplessness and hopelessness was the experience they had in common and from which they needed help and support from professionals at the hospital.

Psychologically, by telling their story, this forms an important basis from where old persons themselves, or together with health care professionals, can loosen the grips and find a way out of their painful existence.
